# An Experimental Study of Hafting Adhesives and the Implications for Compound Tool Technology

**DOI:** 10.1371/journal.pone.0112560

**Published:** 2014-11-10

**Authors:** Andrew M. Zipkin, Mark Wagner, Kate McGrath, Alison S. Brooks, Peter W. Lucas

**Affiliations:** 1 Center for the Advanced Study of Hominid Paleobiology, Department of Anthropology, The George Washington University, Washington, D.C., United States of America; 2 Department of Mechanical and Aerospace Engineering, The George Washington University, Washington, D.C., United States of America; 3 Human Origins Program, Smithsonian Institution, US National Museum of Natural History, Washington, D.C., United States of America; 4 Department of Bioclinical Sciences, Kuwait University, Kuwait City, Kuwait; University of Oxford, United Kingdom

## Abstract

Experimental studies of hafting adhesives and modifications to compound tool components can demonstrate the extent to which human ancestors understood and exploited material properties only formally defined by science within the last century. Discoveries of Stone Age hafting adhesives at archaeological sites in Europe, the Middle East, and Africa have spurred experiments that sought to replicate or create models of such adhesives. Most of these studies, however, have been actualistic in design, focusing on replicating ancient applications of adhesive technology. In contrast, this study tested several glues based on *Acacia* resin within a materials science framework to better understand the effect of each adhesive ingredient on compound tool durability. Using an overlap joint as a model for a compound tool, adhesives formulated with loading agents from a range of particle sizes and mineral compositions were tested for toughness on smooth and rough substrates. Our results indicated that overlap joint toughness is significantly increased by using a roughened joint surface. Contrary to some previous studies, there was no evidence that particle size diversity in a loading agent improved adhesive effectiveness. Generally, glues containing quartz or ochre loading agents in the silt and clay-sized particle class yielded the toughest overlap joints, with the effect of particle size found to be more significant for rough rather than smooth substrate joints. Additionally, no particular ochre mineral or mineral mixture was found to be a clearly superior loading agent. These two points taken together suggest that Paleolithic use of ochre-loaded adhesives and the criteria used to select ochres for this purpose may have been mediated by visual and symbolic considerations rather than purely functional concerns.

## Introduction

Evidence for hafted and composite tools in the Middle Stone Age (MSA) and Middle Palaeolithic (MP) archaeological record is frequently discussed as a potential signature of behavioral complexity or modern behavior, even by researchers with diverse points of view on the concept of “modernity” [1], [2], [3], [4]. The act of hafting, that is the construction of a composite tool from an insert which forms the working edge, a joint, and a handle, has been described as a complex technology. The individual components of such tools are of limited utility on their own, evidencing the cognitive complexity underlying their construction. Barham [5], citing cognitive neuroscientist Scott Frey [6], characterizes hafted tools as complex because they qualitatively alter the actions of our limbs and our perception of a tool as an extension of our arms. Other authors have emphasized the construction of composite tools through additive processes, as opposed to the reductive activity of knapping, and have used such tools to infer the emergence of specific cognitive capabilities including abstract thought, recursion, and multitasking or cognitive fluidity [1], [3]. Ambrose [1] in particular has interpreted hafting through the lens of constructive memory, a proposed subset of working memory that he asserts co-evolved with planning and task coordination abilities.

Controversial evidence for hafted spear tips from the earliest MSA at Kathu Pan 1, South Africa dates back to ∼500 ka [7] but evidence for the use of adhesives in particular is lacking at this site as it can only be identified at the highest level of certainty from residues on artifacts [5]. Numerous examples from MP/MSA and younger contexts have been identified in Europe [8], [9], [10], [11], the Middle East [12], [13], [14], and Africa [15], [16], [17], [18], [19]. Consequently, experimental archaeology studies investigating the formulation and use of adhesives have proliferated in order to interpret this new evidence [20], [21], [22], [23]. These efforts to replicate, or produce useful models of, the earliest glues used to haft lithic tools have largely been actualistic in design and focused on reconstructing adhesive formulation and application. Recently, Charrié-Duhaut and colleagues [15] have asserted that the study of archaeological evidence for hafting technology “represents a discrete area of investigation in its own right that permits access to a lost technological “savoir-faire””. The authors of the study presented here concur and believe that in order to draw generalizable conclusions about the evolution of composite tool technology and the cognitive capabilities that facilitated it, actualistic experimental archaeology should be complemented by narrowly defined studies of the variables that determine hafting adhesive effectiveness. Our study measured the toughness of several model adhesives formulated from *Acacia* resin and ochres of diverse mineralogy and quartz sands of variable particle size in order to better understand the technological requirements of hafting technology, part of what Barham [5] has termed “The First Industrial Revolution”.

A wide range of hafting adhesives have been used worldwide from the MSA/MP through present day, with some of the best known including birch bark tar [11], [20], bitumen/asphaltum [12], [13], [14], amber [24], various conifer resins with and without ochre [25], *Spinifex* resins [26], *Protorhus longifolia* resin [27], and *Acacia* resins [28]. A search of the experimental archaeology literature will yield studies that have employed many of the above adhesives, as well as modern synthetic glues, for the purpose of constructing hafted tools. Most of these investigations focused not on the adhesive itself but rather were testing a hypothesis about projectile armatures; an informative table summarizing thirty years of projectile experiments and the hafting method used in each was included in a recent publication on use-wear analysis [29]. One of the few recent studies to emphasize the hafting method benefitted from an experimental design informed by ethnohistoric accounts of asphaltum use as an adhesive in the Central Valley of California [30]. For researchers interested in studying hafting adhesives in the context of the African Stone Age and human cognitive evolution no such accounts exist and residue evidence supporting the use of a specific adhesive is limited.

Of particular importance to this project are the reports of plant resin residues on Howiesons Poort (HP) segments from 50–64 ka BP at Sibudu Cave, South Africa which also display macrofractures, microwear, and blood residues strongly suggestive of their use as arrow armatures in hunting [17], [18], [31], [32]. Some such tools are also stained with red and yellow ochre; these residues are generally present where the tool would have been attached to a haft, suggesting the incorporation of ochre into the adhesive mixture. The evidence for the use of resin and ochre hafting adhesives at Sibudu, as well as on HP artifacts at Umhlatuzana Rock Shelter (60 ± 4 ka) [33] and Rose Cottage Cave (68–60 ka BP) [16], spurred replication experiments [21], [22], [23] using gums of various *Acacia* species as a model for the residues found on artifacts. The most widely available of these gums, gum arabic, is defined as “a dried exudate obtained from the stems and branches of *Acacia senegal* (L.) Willdenow or *Acacia seyal* (Fam. Leguminosae)” [34]. *Acacia* exudates are variable even within a single species and decades of research have been required to elucidate the structure and composition of commercially important gums [35]. Artisans formulating adhesives would have needed to understand concepts like malleability and toughness to be able to modify such properties and produce a consistently effective product from inconsistent natural ingredients. Pioneering experimental replications of Upper Paleolithic hafting adhesives by Allain and Rigaud [36] demonstrated that adhesives can be improved by adding an inert filler, or loading agent, such as finely ground ochre. The addition of an ochre loading agent to plant resin or a resin and wax mixture has been found to make the resulting adhesive easier to apply, faster drying, less hydroscopic after drying, and ultimately less brittle [21], [22], [23], [36]. Wadley and colleagues [22], as well as Hodgskiss [21], concluded, based on task-oriented experiments with adhesive-hafted tools, that a loading agent containing diverse particle sizes is required to formulate successful glues. The loading agent was proposed to fill a role comparable to the aggregate component of modern concrete. MSA artisans may have been aware of this and deliberately introduced a coarse particle component to improve adhesive toughness. Although many qualities contribute to the effectiveness of an adhesive, bond toughness is both central to the purpose of a glue and a measurable, mathematically defined property. The study presented here was undertaken for the purpose of testing the effects of loading agent mineralogy and particle size on adhesive joint toughness in a more controlled environment than has been used in previous research on this subject.

## Experimental Design

**Figure 1:**
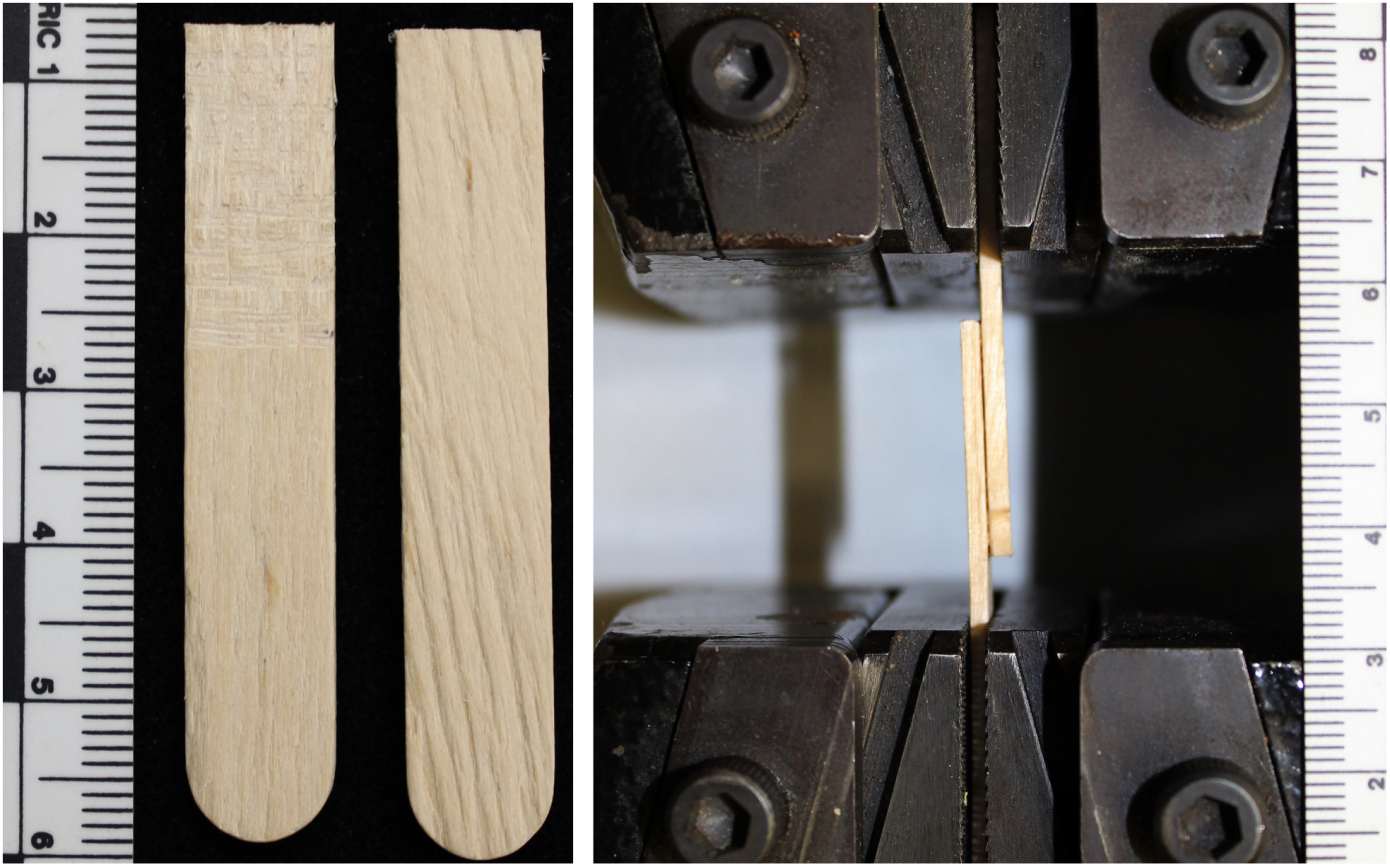
Experimental design for measuring adhesive fracture energy of an overlap joint. Panel 1a shows a roughened wood substrate shown on the left and a smooth (unmodified) substrate on the right. Panel 1b shows a complete overlap joint loaded into the ATS Series 900 Universal Testing Machine.

The previously cited hafting adhesive experiments favored a realistic approach which, while readily applicable to the interpretation of archaeological artifacts, conflated the effects of multiple variables such as adhesive ingredients, drying conditions, and variation in task performance by tool users. The experimental design used here is intended to complement the existing body of research and represents a conscious trade-off between generality, realism, and precision with a bias favoring generalizable and precisely measurable results. The model system (Figure 1a) used to measure the toughness of adhesive compounds consisted of an overlap joint constructed from a wooden substrate with a roughened or unmodified (smooth) surface and adhesives formulated from gum arabic and one of several loading agents. These overlap joints, each of which represented the haft of a compound tool, facilitated the testing of different adhesives in a standardized manner. By making hundreds of copies of the same, very simple, compound tool it was possible to isolate the effects of the adhesive loading agents from many of the other variables that may affect haft durability. Overlap or lap joints are of considerable historical importance, a prominent example being paper production by overlapping papyrus stems in ancient Egypt [37]. In addition, overlap joints are directly relevant to the interpretation of how prehistoric compound tools were constructed and are commonly used in experimental archaeology under the terms “slot haft” and “notch haft”. For example, in the recently published study of the relative lethality of tipped and untipped spears, Wilkins and colleagues used an L-notch haft to attach knapped quartzite points to their spears [38]. An L-notch haft is a single overlap joint analogous to the joint used in this study. Despite their widespread use since prehistory, general principles explaining lap joint behavior are a relatively modern development with lap joint failure by elastic stretching first experimentally demonstrated in 1975 [39]. In the study presented here, each overlap joint was loaded in tension (Figure 1b) until the joint failed and the two pieces of wood detached from each other. The peak load during joint failure was used to calculate the material property called “adhesive fracture energy” (also known as work of adhesion), a thermodynamic measure of adhesive joint toughness [40]. Adhesive fracture energy was interpreted as a measure of glue effectiveness, with greater fracture energies corresponding to a more durable hafted tool.

The project was conducted in two phases; Phase 1 focused on adhesives produced using five ochreous loading agents representing a range of iron contents and mineralogical compositions including specularite, hematite, and goethite with various dilutant minerals (Table 1). Each adhesive was formulated from commercially available dried gum arabic, distilled water, and a loading agent, as described in the Methods. In addition, an adhesive was produced using a quartz (silicon dioxide) loading agent as an iron-free control. A second control adhesive was made without any loading agent at all. Phase 2 used loading agents with a range of particle size distributions in order to observe its influence on adhesive toughness. To isolate the effects of particle size from the chemical effects of loading agent mineral composition, only silicon dioxide was used in Phase 2. Classes of particle size are typically characterized according to systems such as the Wentworth [41] grade scale employed here. New experimental adhesives were formulated using six silicon dioxide loading agents, three containing a mixture of particle sizes and three composed of particles from a specific size class (Table 2). A gum arabic only adhesive was also used to construct control lap joints, as in Phase 1. The results for the silicon dioxide loading agent from Phase 1 were also included in analysis of Phase 2 results for comparison (labeled as Loading Agent #1 in Table 2). The complete data set of adhesive fracture energy results for Phases 1 and 2 may be found in Table S1.

**Table 1:** Properties of loading agents used in Phase 1 overlap joint experiments.

Loading Agent	% MassIronasFe2O3	Major FeMineral	% MassSecondaryMinerals	Particle Size Distribution (%)					
				Very coarse sand(1.0–2.0 mm)	Coarse sand(0.5–1.0 mm)	Medium sand(0.25–0.5 mm)	Fine sand(125–250 µm)	Very fine sand(62.5–125 µm)	Silt + Clay(<62.5 µm)
Coarse GroundSpecularite*	Unknown*	α-Fe_2_O_3_ *	Cu_5_(PO_4_)_2_(OH)_4_ *	15.08	12.08	8.01	8.01	19.14	37.68
			Cu_2_ (PO_4_)(OH)*						
			α-FeO(OH)*						
Hoover PT PrimerNatural Red	97.5	α-Fe_2_O_3_	Al_2_O_3_ = 1	0	0	0	0	0	100
			SiO_2_ = 1.5						
Hoover P3 PrimerNatural Red	80	α-Fe_2_O_3_	SiO_2_ = 9	0	0	0	0	0	100
			Al_2_O_3_ = 6						
			MgO = 1						
			CaO = 1						
Hoover GRD NaturalYellow	95–96	α-FeO(OH)	SiO_2_ = 4–5	0	0	0	0	0	100
Hoover GRD548OCH	57–60	α-FeO(OH)	SiO_2_ = 21–24	0	0	0	0	0	100
			H_2_Mg_3_(SiO_3_)_4_ = 3–4						
			MgCO_3_ = 2.5–3.5						
Sigma-Aldrich SiliconDioxide #S5631	0	None	SiO_2_ = 100	0	0	0	0	0	99∧

*Mineral composition was reported by the manufacturer for all loading agents except Coarse Specularite which was produced in-house and analyzed using a Rigaku D/Max Rapid Micro X-ray Diffractometer with AreaMax 2.0 and JADE 8.0 software. The major phase detected by the XRD analysis was identified as the Major Fe Mineral and the minor phases as the Secondary Minerals.

∧100% of particles were silt and clay-sized based on sieving but the manufacturer reported 1% colloidal particles.

**Table 2:** Grain size distribution (% mass) of loading agents used in Phase 2.

Loading Agent†	Source and Note	Coarse Sand (0.5–1.0 mm)	Medium Sand (0.25–0.5 mm)	Fine Sand (125–250 µm)	Very Fine Sand (62.5–125 µm)	Silt + Clay (<62.5 µm)	Loading Agent Particle Size Rank 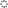
#1: Silt and Clay‡	Sigma-Aldrich reports ∼1% colloidal (<1 µm) particles.	0	0	0	0	99	1
#2: Medium Sand Mix	Hebei Yunsong Trade Co.	20.2	71.6	7.7	0.14	0.36	5
#3: Fine Sand Mix	Hebei Yunsong Trade Co.	0	0.26	80.79	18.71	0.24	3
#4: Silt and Clay Mix	Hebei Yunsong Trade Co.	0	0	0.2	2.05	97.75	2
#5: Medium Sand Only	Separated from #2.	0	100	0	0	0	6
#6: Fine Sand Only	Separated from #2.	0	0	100	0	0	4
#7: Coarse Sand Only	Separated from #2.	100	0	0	0	0	7

† All materials were purchased from commercial suppliers and reported as 100% Silicon Dioxide by their manufacturer.

‡ #1 is the same loading agent as Silicon Dioxide #S5631 in Table 1.

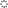
Ranked in ascending order from finest to coarsest by percent of each particle size class represented in the loading agent.

## Materials and Methods

Adhesives were formulated to be generally representative of hafting glues that could have been produced from mineral and vegetal ingredients available in Middle Stone Age Africa and were not intended to be recreations of any specific formulas. Previous experiments with gum arabic adhesives indicated that poorly mixed, heterogeneous glue can confound the interpretation of results [21]. One solution suggested by Hodgskiss [21], which was applied here, was to use dry, finely ground gum reconstituted with water to the desired consistency. Each loading agent-containing adhesive was formulated according to a recipe of 2.5 g of spray dried gum arabic (Acros Organics, Gum Arabic, Catalog #AC258850010) mixed with 2.5 ml of distilled water over a direct heat source adjusted as necessary and stirred until fully dissolved. To this glue base, 1 g of the appropriate loading agent was added and manually stirred until dispersed. The resin-only control was made using a ratio of 1 g gum arabic:1 ml distilled water. The glue was then immediately applied to a 2 cm×0.95 cm area on two pieces of birch wood, each 0.2 cm thick (Figure 1a). The glue-covered areas were then overlapped to create the joint, clamped with a binder clip, and allowed to cure for 48 hours at 40° Celsius. Such a lap joint replicates the same ‘sliding’ conditions that would cause the failure of a hafted joint. For approximately two out three lap joints produced, both pieces of wood substrate were used in their purchased form; this is referred to as the ‘smooth condition substrate’ (Figure 1a right). One out of three joints was constructed using the ‘roughened condition substrate’ where the wood was abraded in a crosshatch pattern over the 2 cm×0.95 cm area intended for overlap using a Dremel rotary tool (Figure 1a left). The elastic modulus *E* of the birch wood substrate was experimentally measured with a 4-point bending test using a Lucas Scientific FLS-I Material Tester and determined to be 9.7 GPa.

Testing of each lap joint was carried out using an Applied Test Systems (ATS) Series 900 Universal Testing Machine. Each joint was inserted into the ATS 900 with the two ends of the joint secured using spring-loaded clamps (Figure 1b). Lap joints were loaded in tension at a constant crosshead speed of 0.05 mm/second. The load on the joint was monitored in real time until joint failure was detected through a precipitous drop in load, sometimes accompanied by audible and visible cracking. Any joints which were perceived to have failed, but on removal from the tester found to still be intact, were assumed to have experienced either incomplete cracking or clamp slippage and were excluded from data analysis. No initial ‘seed’ crack was introduced in the specimens. Nevertheless, it was possible to use the peak load *F* (in Newtons) measured during each experiment to calculate adhesive fracture energy *W* (in Joules/meter^2^) from
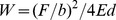
(1)where *b* was the joint breadth (0.0095 meters), *E* was the measured elastic modulus of the wood (9.7 GPa), and *d* was joint depth (0.002 meters) [39]. All statistical analyses were performed in JMP 11 (SAS Institute Inc.) except for the Monte Carlo analysis which was run in R version 3.0.3 (R Foundation for Statistical Computing).

## Results from Phase 1 Experiments: Ochre Loading Agents

**Figure 2:**
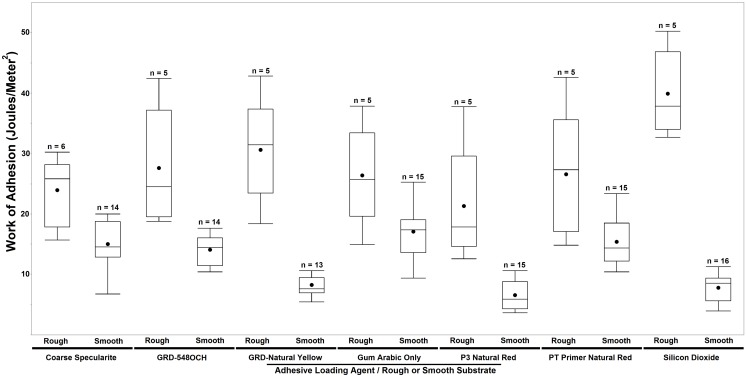
Box and whisker plots of Phase 1 results by adhesive loading agent and substrate condition classes. The horizontal line in each box indicates the median adhesive fracture energy for that class and the solid dot indicates the mean adhesive fracture energy for that class.

In Figure 2 the adhesive fracture energy distributions for all lap joints tested in Phase 1 (N_Phase 1_ = 138) are plotted in classes by adhesive formula and by whether the wood substrate was smooth or roughened. An analysis of adhesive fracture energy results from the lap joint experiments (classified as in Figure 2) was performed using a Tukey-Kramer Honest Significant Difference (HSD) test to identify similarities and differences in adhesive toughness. The Tukey-Kramer HSD test is a multiple comparison procedure used to simultaneously compare all possible pairs of means from a data set with multiple groups; in effect it is a t-test that corrects for the increase in Type I error caused by performing multiple t-tests. The HSD test results indicated significant differences among the loading agents and substrate conditions (q* = 3.421, α = 0.05); complete results are presented in Table 3. These paint a complex picture of which a few major points are addressed here. Overall, the lap joint class that yielded the highest mean adhesive fracture energy (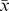
_SiO2, Rough_ = 39.89 J/m^2^) was constructed with the quartz-containing glue applied to a roughened substrate. This class was significantly different from all others with the exception of the GRD-Natural Yellow adhesive, also applied to a rough substrate (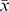
_GRD-NY, Rough_ = 30.61 J/m^2^). This attains greater importance when compared to the three classes that form the weakest joints, all on smooth substrates: GRD-Natural Yellow (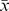
_GRD-NY, Smooth_ = 8.30 J/m^2^), quartz (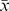
_SiO2, Smooth_ = 7.79 J/m2), and P3 Natural Red (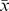
_P3-NR, Smooth_ = 6.54 J/m^2^). The two loading agents with the greatest mean adhesive fracture energies on roughened wood (Quartz and GRD-Natural Yellow) also yielded significantly lower adhesive fracture energies results on a smooth substrate, illustrating that adhesive efficacy is highly dependent on how the joint surface is prepared. In addition, the fact that a silicate loading agent, an iron oxyhydroxide loading agent (GRD-Natural Yellow), and an iron oxide loading agent (P3-Natural Red) are all among the weakest loading agents suggests, though does not prove, that loading agent mineralogy is not a major determinant of gum arabic adhesive performance.

**Table 3:** Results of Tukey-Kramer Honest Significant Difference test of all Phase 1 results°.

Overlap Joint Class byAdhesive Loading Agentand Substrate Condition	Group A	Group B	Group C	Group D	Group E	Group F	Mean AdhesiveFracture Energy(Joules/Meter^2^)	# OverlapJoints (n)
Silicon Dioxide - Rough Substrate	A						39.89	5
GRD-Natural Yellow - Rough Substrate	A	B					30.61	5
GRD-548 - Rough Substrate		B					27.59	5
PT Primer-NaturalRed - Rough Substrate		B					26.54	5
Gum Arabic Only - RoughSubstrate		B					26.36	5
Coarse Specularite - RoughSubstrate		B	C				23.93	6
P3-Natural Red - RoughSubstrate		B	C	D			21.27	5
Gum Arabic Only - SmoothSubstrate			C	D			17.07	15
PT Primer-Natural Red -Smooth Substrate				D			15.39	15
Coarse Specularite - SmoothSubstrate				D			15.03	14
GRD-548 - SmoothSubstrate				D	E		14.09	14
GRD-Natural Yellow -Smooth Substrate					E	F	8.30	13
Silicon Dioxide - SmoothSubstrate						F	7.79	16
P3-Natural Red - SmoothSubstrate						F	6.54	15

°Classes not connected by the same letter are significantly different. q* = 3.421, α = 0.05, N = 138.

For all adhesives tested in Phase 1, joints with roughened substrates always yielded higher mean adhesive fracture energies than those with smooth substrates, most likely due to the increased surface area to which the glue layer could adhere. When results for all adhesives (N = 138) were pooled and grouped only by substrate (n_smooth_ = 102; n_rough_ = 36), the roughened group exhibited a significantly greater mean adhesive fracture energy (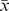
_rough_ = 27.91 J/m^2^) than the smooth wood group (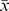
_smooth_ = 12.01 J/m^2^) according to Student’s *t*-test (*t* = 1.978, α = 0.05, p<0.0001) and a Mann-Whitney-Wilcoxon test (Normal Approximation, *z* = 7.857, α = 0.05, p<0.0001). This is readily apparent in Figure 2 where, for any given glue, the rough substrate lap joint class exhibited higher median adhesive fracture energy than smooth joints assembled with that same glue.

## Results from Phase 2 Experiments: Variable Particle Size Silicon Dioxide Loading Agents

**Figure 3:**
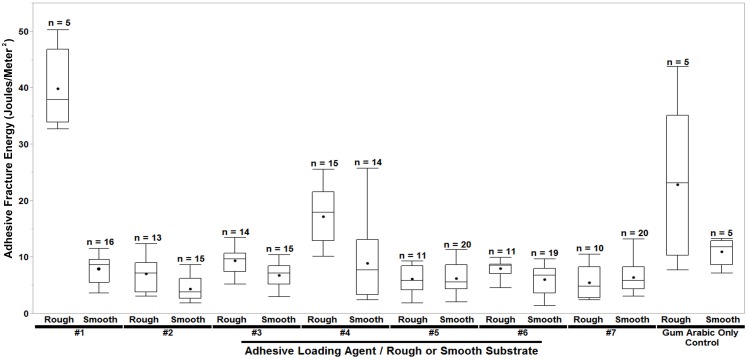
Box and whisker plots of Phase 2 results by adhesive loading agent and substrate condition classes. The horizontal line in each box indicates the median adhesive fracture energy for that class and the solid dot indicates the mean adhesive fracture energy for that class.

The Phase 2 experiments, which used only silicon dioxide loading agents, sought to determine the effect of loading agent particle size on overlap joint adhesiveness. Figure 3 illustrates the distribution of adhesive fracture energies for each adhesive and substrate class in Phase 2 (N_Phase 2_ = 208). A statistical analysis of the entire Phase 2 data set was performed using a Tukey-Kramer HSD test with results divided as shown in Figure 3. As previously noted, the results for the silicon dioxide loading agent from Phase 1 (referred to as loading agent #1 in Phase 2) were included in this analysis for comparative purposes. The Tukey-Kramer HSD (q* = 3.472, α = 0.05, full results in Table 4) identified loading agent #1 on a roughened substrate as the class of lap joint which yielded a mean adhesive fracture energy (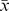
_#1, Rough_ = 39.89 J/m^2^) significantly greater than all others tested. The next strongest group was gum arabic only adhesive on a rough substrate (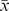
_Gum Arabic Only, Rough_ = 22.84 J/m^2^) and loading agent #4 (silt and clay-sized particles with very fine sand) on a rough substrate (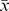
_#4, Rough_ = 17.15 J/m^2^). These two lap joint classes yielded statistically-comparable mean adhesive fracture energies but significantly lower means than loading agent #1. All other joint classes were generally indistinguishable from one another in performance and weaker than the above noted classes.

**Table 4:** Results of Tukey-Kramer Honest Significant Difference test of all Phase 2 results^♣^.

Overlap Joint Class by Adhesive LoadingAgent and Substrate Condition	Group A	Group B	Group C	Group D	Group E	Mean AdhesiveFracture Energy(Joules/Meter^2^)	# OverlapJoints (n)
#1, Silt and clay-sized with colloidal particles– Rough Substrate	A					39.89	5
Gum Arabic Only – Rough Substrate		B				22.84	5
#4, Silt and clay-sized with very fine sand –Rough Substrate		B	C			17.15	15
Gum Arabic Only – Smooth Substrate			C	D	E	10.99	5
#3, Fine sand mixture – Rough Substrate				D		9.38	14
#4, Silt and clay-sized with very fine sand –Smooth Substrate				D	E	8.93	14
#6, Fine sand only – Rough Substrate				D	E	7.99	11
#1, Silt and clay-sized with colloidal particles– Smooth Substrate				D	E	7.79	16
#2, Medium sand mixture – Rough Substrate				D	E	7.05	13
#3, Fine sand mixture – Smooth Substrate				D	E	6.82	15
#7, Coarse sand only – Smooth Substrate				D	E	6.38	20
#5, Medium sand only – Smooth Substrate				D	E	6.19	20
#5, Medium sand only – Rough Substrate				D	E	6.17	11
#6, Fine sand only – Smooth Substrate				D	E	6.06	19
#7, Coarse sand only – Rough Substrate				D	E	5.45	10
#2, Medium sand mixture – Smooth Substrate					E	4.33	15

^♣^Classes not connected by the same letter are significantly different. q* = 3.472, α = 0.05, N = 208.

In order to further interpret the effects of particle size, Spearman’s rank correlation coefficients (ρ) were calculated for the percentage of each Wentworth particle size class present in the loading agents and the adhesive fracture energies associated with those loading agents. Using the particle size distributions in Table 2, correlations were calculated separately for the rough and smooth joints with the gum arabic only control joints excluded (Table 5). For the smooth condition (n_Smooth, Excluding Control_ = 119), the only significant correlation (α = 0.05) was for coarse sand (ρ = −0.1847), though all correlations steadily changed from negative towards positive with decreasing particle size. The only positive correlation found, for the silt and clay-sized particle class, was not significant (ρ = 0.1067). When the rough substrate results (n_Rough, Excluding Control_ = 79) were examined, once again, Spearman’s ρ became increasingly positive across particle size classes in order of decreasing particle size. In contrast to the smooth lap joint results, for the rough substrate results significant correlations were found for all particle size classes except fine sand-sized particles.

**Table 5:** Spearman’s rank correlation of particle size and Adhesive Fracture Energy for Phase 2 results§.

Particle Size Class	Spearman ρ for Correlation of %Particle Size Class with AdhesiveFracture Energy for SmoothOverlap Joints	Probability >|ρ|for SmoothOverlap Joints(n = 119)	Spearman ρ for Correlation of %Particle Size Class with AdhesiveFracture Energy for RoughenedOverlap Joints	Probability >|ρ|for RoughOverlap Joints(n = 79)
% CoarseSand	−0.1847	0.0443	−0.4583	<.0001
% MediumSand	−0.1705	0.0637	−0.3924	0.0003
% FineSand	−0.1033	0.2634	0.0191	0.8675
% VeryFine Sand	−0.0390	0.6738	0.3548	0.0013
% Silt andClay	0.1067	0.2480	0.6658	<.0001

§ Correlations calculated separately for roughened and smooth joints. Gum arabic only control joints (n = 10) were excluded from the calculations.

While the authors believe that this analysis is useful, there are weaknesses associated with using rank correlation on a data set where the independent variables exhibit a limited range of values (*e.g.* Coarse Sand may only equal 0%, 20.2%, or 100%); many ties will occur in the ranking process and result in unreliable p-values. To address this issue, each loading agent was first ranked from finest to coarsest (Table 2) according to the percentage of each particle size class represented in the mixture. Spearman’s coefficients (ρ) were then calculated for adhesive fracture energy values and the rank of the loading agent used; this provided a relatively crude but robust measure of the correlation between particle size and adhesive toughness. A Monte Carlo analysis was then performed using the Spearman’s correlation. Specifically, the observed ρ was compared to 10,000 iterations where adhesive fracture energy was randomized (sampled without replacement) while ranks were held constant. The p-value in this case was the probability of the simulation producing the same or a more extreme result than the observed ρ. That analysis found ρ = −0.7750 and p<0.0001 for the roughened joints and ρ = −0.1991 and p = 0.0320 for the smooth joints, further substantiating the apparent relationship between particle size and fracture energy and the difference in sensitivity to particle size between smooth and rough substrates.

## Analysis of Combined Phase 1 and Phase 2 Results

Thus far the Phase 1 and Phase 2 results have been examined separately; lastly a combined analysis of both phases was run. This is perhaps better for interpreting the decision-making process of an individual constructing a compound tool since the loading agents used here were intended as proxies for widely available natural materials: quartz sand of various particle sizes and ochres of diverse mineralogy. A Tukey-Kramer HSD test was done separately for all smooth and rough joints from both phases (complete results in Table 6 and Table 7). For the smooth substrates, the group of the four toughest adhesives was composed of the gum arabic control and the adhesives containing the ochres PT Primer Natural Red, Coarse Specularite, and GRD-548OCH. For the rough substrates, loading agent #1, the silt and clay-sized quartz, was found to yield the greatest mean fracture energy, although this was not significantly different from lap joints constructed with ochres GRD-Natural Yellow or GRD-548OCH, both of which were mostly composed of goethite. A general result is that all ochre-loaded adhesives outperformed all of the quartz-containing adhesives, with the notable exception of loading agent #1.

**Table 6:** Results of Tukey-Kramer Honest Significant Difference test of all Roughened Substrate Joint results from Phases 1 and 2^
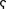
^.

Overlap Joint Class byAdhesive Loading Agent	Group A	Group B	Group C	Group D	Mean AdhesiveFracture Energy(Joules/Meter^2^)	# Overlap Joints (n)
#1, Silt and clay-sized withcolloidal particles	A				39.89	5
GRD-Natural Yellow	A	B			30.61	5
GRD-548OCH	A	B			27.59	5
PT Primer Natural Red		B	C		26.54	5
Gum Arabic Only Control		B	C		24.60	10
Coarse Specularite		B	C		23.93	6
P3 Natural Red		B	C		21.27	5
#4, Silt and clay-sized withvery fine sand			C		17.15	15
#3, Fine sand mixture				D	9.38	14
#6, Fine sand only				D	7.99	11
#2, Medium sand mixture				D	7.05	13
#5, Medium sand only				D	6.17	11
#7, Coarse sand only				D	5.45	10

^
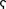
^ Classes not connected by the same letter are significantly different. q* = 3.39273, α = 0.05, N = 115.

**Table 7:** Results of Tukey-Kramer Honest Significant Difference test of all Smooth Substrate Joint results from Phases 1 and 2^
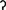
^.

Overlap Joint Class by AdhesiveLoading Agent	Group A	Group B	Group C	Mean AdhesiveFracture Energy(Joules/Meter^2^)	# Overlap Joints (n)
Gum Arabic Only Control	A			15.55	20
PT Primer Natural Red	A			15.39	15
Coarse Specularite	A			15.03	14
GRD-548OCH	A			14.09	14
#4, Silt and clay-sized withvery fine sand		B		8.93	14
GRD-Natural Yellow		B	C	8.30	13
#1, Silt and clay-sized withcolloidal particles		B	C	7.79	16
#3, Fine sand mixture		B	C	6.82	15
P3 Natural Red		B	C	6.54	15
#7, Coarse sand only		B	C	6.38	20
#5, Medium sand only		B	C	6.19	20
#6, Fine sand only		B	C	6.06	19
#2, Medium sand mixture			C	4.33	15

^
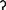
^Classes not connected by the same letter are significantly different. q* = 3.35400, α = 0.05, N = 210.

## Discussion

Firstly, roughening the surfaces of a haft prior to the application of adhesive is an extremely effective way of increasing lap joint toughness. A qualification is that the calculation of adhesive fracture energy for the single material (wood) lap joint used here is dependent on the elastic modulus of that material. As a consequence, conclusions regarding the effects of substrate roughening may not be transferrable to composite tools constructed from a lithic insert hafted onto wood. However, our results are supported by theoretical models of fracture in bimaterial interfaces in which roughness of the interface crack surface causes crack shielding and can lead to markedly higher fracture energy values [42]. Subsequent experimental studies of compound tool construction can control for and examine the effects of roughness amplitude and directionality which were outside the scope of this investigation. Abrading or incising the haft surfaces of composite tools was certainly within the technological capabilities of Stone Age humans and examining such modifications on archaeological artifacts may shed light on an ancient comprehension of material properties that were only recently defined formally. Archaeological evidence for roughening hafting surfaces can be found in the form of scratches and incisions on obsidian Clovis (∼13.2–12.9 ka BP) points [24], [43] and on bone and ivory Clovis foreshafts [44]; these have speculatively been interpreted as a means of toughening a haft [24]. Similar modifications have been identified on *sagaie* points from the Upper Paleolithic Magdalenian culture (∼17–12 ka BP) of Western Europe, which may have functioned as a spear or harpoon armature [45]. The utility of such modifications for fixing an armature to a handle have long been noted [36] and this interpretation echoed by more recent authors [45]. Residues tend to become trapped in such incisions [36], suggesting an avenue for studies of ancient adhesive composition.

While overlap joint toughness was improved overall by roughening there is also evidence that roughening makes lap joints more sensitive to the particle size distribution of the loading agent. This is strongly suggested by the correlations (Table 5) calculated for particle size and adhesive fracture energy using the Phase 2 data and the finding that the correlations become positive as particle sizes are decreased. The same relationship was identified irrespective of joint roughness. However, most correlations were not significant for the smooth condition, whereas for the rough joints, the correlations (ρ) were of greater magnitude and nearly all significant. This interpretation is also supported by the Monte Carlo analysis of the correlation between fracture energy and loading agent particle size rank, which found a far weaker relationship for smooth joints than roughened joints.

In the combined Phase 1 and 2 data analysis for roughened joints (Table 6), the ochre-loaded adhesives had higher mean fracture energies than the quartz-loaded adhesives, except for quartz #1 which yielded the toughest glue overall. All ochre loading agents except for Coarse Specularite are composed of 100% silt and clay-sized particles, a factor that they have in common with quartz #1. This further suggests that on a rough surface, the silt and clay particle size class of the ochres and not their mineralogy is the basis of adhesive effectiveness. However, this explanation raises the question of why the Coarse Specularite loaded glue behaved comparably to the other finer and more uniform ochres. It is possible that, despite being rather coarse grained, the platy crystal structure of specularite caused it to behave more like the other clay and silt-sized ochres. Loading agent particle shape and angularity were not controlled for here and should be addressed in future research. The overall pattern found in Phase 2, where rough substrates were more sensitive to particle size than smooth substrates, may explain in part why there is no evidence for the roughening or incising of hafted lithic inserts at sites like Sibudu. Although roughening can dramatically improve the toughness of an adhesive bond it also might necessitate more stringent processing or selection of adhesive ingredients.

A converse interpretation of these results is also worth considering. By using a smooth and deliberately shaped piece of wood for the handle with a lithic insert made of a smooth fine-grained rock, the haft would likely be less sensitive to the loading agents, which could reduce the labor exerted during intensive grinding or the acquisition of a suitable agent from a specific desirable source. Furthermore, for the smooth substrate, the joints glued with gum arabic only yielded the highest mean adhesive fracture energy. Thus, the construction of a hafted tool from smooth rock and plant materials could facilitate both the use of a wider range of loading agent particles sizes or else a single component adhesive with no loading agent at all. This point is speculative and additional experimental research using lap joints constructed of a wooden substrate overlapped with a lithic material is required to test it. However, in light of these findings, we can take a new look at some existing research regarding resin and ochre hafted tools from the African MSA.

In the Supplementary Information to Wadley and colleagues’ experimental study [22], the authors note that, for the HP assemblage from Sibudu Cave, South Africa, the backed segments made on quartz preserve residue of plant resin alone more often that they preserve resin and ochre. This point is based on a small sample [46], but the same pattern appears for quartz segments from the HP at Umhlatuzana Rock Shelter, South Africa. Plant resin occurred 269 times on 25 quartz segments from Umhlatuzana, but with only 43 occurrences of ochre [33]. This contrasts with approximately equivalent representation of ochre (238) and resin (269) residues on 30 non-quartz segments at the same site. Anticipating the results of the study presented here, the authors noted that “It is therefore feasible to consider that a different, possibly ‘‘stickier’’ adhesive recipe may have been used for hafting quartz tools” [33]. That different recipe may well have been one with little or no loading agent at all since ochre-loaded resin is more easily manipulated but less sticky than resin alone [23]. Future interpretations of archaeological assemblages preserving adhesive residues may be able to take into account the surface roughness of the residue-bearing lithics. More generally though, it should be a central consideration of future studies that there is not a single optimal glue recipe; adhesive technology might best be viewed as a suite of alternative strategies dependent on available materials and the ultimate intended use of the tool.

## Conclusions

In addition to the well-developed cognitive archaeology arguments of Wadley and colleagues regarding the formulation of adhesive from multiple ingredients, studying *how* adhesives were applied presents a new avenue of investigation into the cognitive requirements for making compound tools. The study presented here indicates that the condition of the joint surface can fundamentally alter the effectiveness of a glue and that this variable can be more strongly emphasized in future interpretations of adhesive-hafted tools. The fact that a roughened joint surface results in a tougher bond seems so intuitive that it is hardly worth noting, and yet this point has not featured prominently in previous experimental studies of hafting adhesives. Compared to the conclusions of Wadley and colleagues [22] and Hodgskiss [21] no evidence was found to suggest that loading agents with a mixture of particle sizes result in more effective adhesives than those of a single particle size class, though this study addressed only bond toughness as a proxy for adhesive effectiveness. In particular, the results indicate that loading agents ground to uniformly silt and clay-sized particles, such as most of the ochres and quartz loading agent #1, perform especially well on rough surfaced joints. Particle size is indeed a major determinant of adhesive effectiveness; however this study suggests that if finely ground quartz can perform just as well as or better than finely ground ochres, then the decision to use ochre loading agents in antiquity may have been mediated, at least in part, by symbolic concerns. Actualistic studies are a valuable and necessary starting point for such investigations since they allow researchers to gain experiential insight into the “lost technological savoir-faire” of hafting adhesives [15]. The further development of middle-range theory for interpreting archaeological evidence for such adhesives and compound tool construction in general, however, requires more narrowly controlled experiments which can identify the mechanisms underlying the observations made in actualistic and replicative studies.

## Supporting Information

Table S1 **Adhesive fracture energy values for every overlap joint included in the statistical analyses described in this article.** Note that overlap joint numbers are non-sequential because any joints that were damaged during insertion into the universal testing machine or that experienced clamp slippage during loading were discarded and not included in the analyses.
(XLSX)
